# A Honey Bee Hexamerin, HEX 70a, Is Likely to Play an Intranuclear Role in Developing and Mature Ovarioles and Testioles

**DOI:** 10.1371/journal.pone.0029006

**Published:** 2011-12-19

**Authors:** Juliana R. Martins, Lucas Anhezini, Rodrigo P. Dallacqua, Zilá L. P. Simões, Márcia M. G. Bitondi

**Affiliations:** 1 Departamento de Genética, Faculdade de Medicina de Ribeirão Preto, Universidade de São Paulo, Ribeirão Preto, São Paulo, Brasil; 2 Departamento de Biologia Molecular e Celular e Bioagentes Patogênicos, Faculdade de Medicina de Ribeirão Preto, Universidade de São Paulo, Ribeirão Preto, São Paulo, Brasil; 3 Departamento de Biologia, Faculdade de Filosofia, Ciências e Letras de Ribeirão Preto, Universidade de São Paulo, Ribeirão Preto, São Paulo, Brasil; University of Otago, New Zealand

## Abstract

Insect hexamerins have long been known as storage proteins that are massively synthesized by the larval fat body and secreted into hemolymph. Following the larval-to-pupal molt, hexamerins are sequestered by the fat body via receptor-mediated endocytosis, broken up, and used as amino acid resources for metamorphosis. In the honey bee, the transcript and protein subunit of a hexamerin, HEX 70a, were also detected in ovaries and testes. Aiming to identify the subcellular localization of HEX 70a in the female and male gonads, we used a specific antibody in whole mount preparations of ovaries and testes for analysis by confocal laser-scanning microscopy. Intranuclear HEX 70a foci were evidenced in germ and somatic cells of ovarioles and testioles of pharate-adult workers and drones, suggesting a regulatory or structural role. Following injection of the thymidine analog EdU we observed co-labeling with HEX 70a in ovariole cell nuclei, inferring possible HEX 70a involvement in cell proliferation. Further support to this hypothesis came from an injection of anti-HEX 70a into newly ecdysed queen pupae where it had a negative effect on ovariole thickening. HEX 70a foci were also detected in ovarioles of egg laying queens, particularly in the nuclei of the highly polyploid nurse cells and in proliferating follicle cells. Additional roles for this storage protein are indicated by the detection of nuclear HEX 70a foci in post-meiotic spermatids and spermatozoa. Taken together, these results imply undescribed roles for HEX 70a in the developing gonads of the honey bee and raise the possibility that other hexamerins may also have tissue specific functions.

## Introduction

The larvae of holometabolous insects accumulate a large quantity of proteins, carbohydrates and lipids which serve as energy and structural compounds for sustaining metamorphosis up to the adult stage [1]. The most abundant proteins in larval hemolymph are the hexamerins, also known as larval serum proteins, or simply, as storage proteins. Hexamerins are high molecular mass molecules composed, by definition, of six subunits, which can be either homo- or heteromers. Evolutionarily they are derived from hemocyanins, but in contrast to the ancestral molecule, they have lost the capacity of binding copper ions for oxygen transport, and mainly have a role as storage proteins [2].

Hexamerins are massively synthesized by the larval fat body and secreted in hemolymph. Following cessation of larval feeding in preparation to the larval-to-pupal molt, these proteins are sequestered from hemolymph by the fat body cells, via endocytosis mediated by membrane receptors [3], and stored in the cytoplasm in the form of granules [4]. As such, they can be processed and used as amino acid source for development completion. In line with the idea that the sole function of most hexamerins is to act as amino acid reserves when feeding is no longer occurring, as during the pupal and pharate-adult stages, Roberts and Brock (1981) [5] considered that hexamerins are the essential proteins for metamorphosis, as vitellogenins are to embryogenesis.

The importance of hexamerins as amino acid storage proteins during metamorphosis was initially demonstrated by injecting larvae of the dipteran *Calliphora vicina* with [^14^C]-phenylalanine that was metabolically incorporated into hexamerin molecules (then called calliphorins), and following the fate of the radioactive carbon isotope. Using this strategy, Levenbook and Bauer (1984) [6] verified that most of the soluble proteins from practically all tissues of the developing pharate-adults became labeled. In a similar experiment, labeled proteins were recorded not only in adult somatic tissues (integument, thoracic muscle), but also in the egg (chorion, yolk) of *Actias luna*, a moth that produces its eggs during pharate adult development [7]. A correlation between egg production and depletion of the larval reserve of hexamerins was established in adult lepidopterans unable to eat (without mouth parts) or that feed basically on nectar, a poor protein diet [7]–[10] despite containing amino acids of supplemental nutritional value [11]. There is also circumstantial evidence that amino acids held in hexamerins are used for provisioning eggs of non-lepidopteran species, such as, the mosquito *Aedes atropalpus*, which produces the first batch of eggs without a feeding [12], [13], the cockroach *Blaberus discoidalis* [14], the house fly *Musca domestica* [15], and the grasshopper *Schistocerca americana* [16]. The high level of hexamerins stored by *Camponotus festinatus* queen ants and by certain species of termites was also related to the production of the first batch of brood without access to food during colony founding [17]–[19]. Together, these results indicate that hexamerin residues are recycled to make other proteins needed for tissues reconstruction during metamorphosis and, in some insect species, for egg production. Thus, after hexamerin breakdown in the fat body, the released amino acid residues are reutilized and incorporated into new proteins, although there is also evidence of incorporation of hexamerins into tissues after partial degradation [20] or even without degradation [4], [21].

In general, hexamerins disappear from hemolymph within a few days after adult eclosion. Nevertheless, in some insect species they may persist in hemolymph up to the adult stage [14], [22]. There is also evidence of synthesis reinduction and even *de novo* synthesis in adults, although at a lower rate [13], [23].

A special class of hexamerins, the arylphorins, has received special attention in view of their high content of aromatic amino acids. In fact, arylphorins have long been presumed to be a source of aromatic amino acids for exoskeleton sclerotization in lepidopterans [7], [24]–[27]. Hexamerins from *Locusta migratoria* [28], [29] and *Melanoplus sanguinipes* [30] also play a role as hemolymph juvenile hormone transporters, and the Larval Hemolymph Protein-1 of *Calliphora vicina* has been confirmed as a low affinity carrier protein for ecdysteroids [4]. Recently, Zalewska *et al.* (2009) [31] demonstrated that hexamerins interact with other proteins (juvenile hormone binding protein and apolipophorin) in a multiprotein complex engaged in sequestration and transport of juvenile hormone, thus inferring the involvement of hexamerins in regulating juvenile hormone levels and action, even when they do not directly bind to the hormone.

Based on the purported ability of binding and controlling juvenile hormone levels, hexamerins have been linked to important facets of social insect life histories. In the termite *Reticulitermes flavipes*, the role of hexamerins has been associated to the regulation of the juvenile hormone-dependent soldier caste phenotype [32]–[35]. Also in honey bee larval development, the inverse relationship between the levels of hexamerin transcripts in the fat body and the juvenile hormone titer suggests that hexamerins may act as players in the juvenile hormone-dependent differentiation of the bipotent female larva towards a queen or a worker phenotype [36]. In the social wasp *Polistes metricus*, one hexamerin may be involved in caste-specific behaviors and in the regulation of diapause, which is also conditional on a low titer of juvenile hormone [37].

Except for the termite *R. flavipes*, most of the above mentioned considerations on the roles of hexamerins in social insect life histories are based on correlational or other circumstantial evidence, still requiring experimental confirmation and in-depth analysis at the cellular level.

In the highly eusocial honey bee, Ryan *et al.* (1984) [38] were the first to characterize a hexamerin subunit in the range of 75–80 kDa. Later, four hexamerin subunits (including the one previously described by Ryan [38]) were distinguished in honey bee hemolymph samples by SDS-PAGE and N-terminal sequencing [39]. Since three of these subunits presented molecular mass in the 70 kDa range, they were named HEX 70a, HEX 70b, and HEX 70c. The other subunit migrated at a rate consistent with a higher molecular mass and was named HEX 110. Studies undertaken in our laboratory led to the characterization of the full-length cDNAs encoding the four honey bee hexamerin subunits. These studies enabled the characterization of the structure of these genes and the prospection of overrepresented sequence motifs indicative of mutual co-regulation in the respective upstream control regions. It was also investigated the evolutionary relationship between the honey bee hexamerins and homologous proteins from other insect species. Furthermore, we characterized the expression patterns of the four hexamerin genes in the fat body and gonads of developing and adult workers, queens and drones, as well as the hormonal- and nutritional- dependent expression of these genes [23], [36], [40], [41].

A honey bee arylphorin, HEX 70a, is the focus of the current work. Through RT-PCR (semiquantitative and quantitative) and western blot analyses using a specific antibody we had previously demonstrated that, besides being strongly expressed in the larval fat body, the HEX 70a transcript and protein subunit were also present in the male and female gonads [23]. In the search for a role of this hexamerin in ovaries and testes we designed experiments for its immunofluorescence detection by confocal laser-scanning microscopy. In parallel, a nucleoside analog of thymidine coupled to a dye was used for prospection of dividing cells in developing ovaries. To highlight structural aspects of the gonads at the developmental stages here approached we used rhodamine-phalloidin labeling for F-actin and DAPI-labeling for cell nuclei, in addition to conventional histology.

## Results

### HEX 70a detection in ovarian cell nuclei in pharate-adult workers

**Figure 1:**
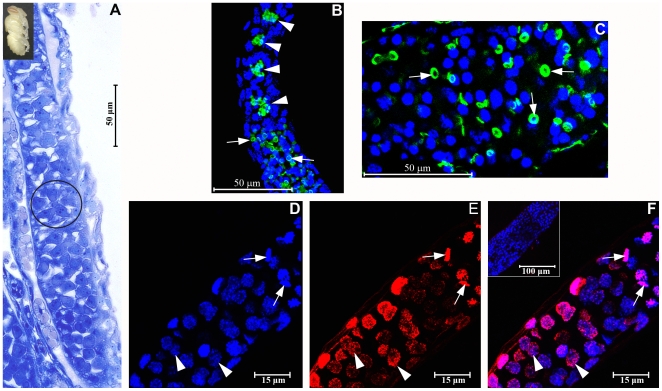
Detection of HEX 70a in ovarioles of workers at the beginning of the pharate-adult development (∼1 day after pupal ecdysis) (the developmental stage is illustrated at the upper left corner of the figure). (**A**) Light microscopy of ovarioles (covered by their respective peritoneal sheath) stained with methylene blue/basic fuchsin. Only the germarium is focused in this figure (the most anterior region of the ovariole, or terminal filament, is not shown). A rosette formed by germline cells (oocyte and nurse cell precursors) is distinguishable (circle) in the germarium. (**B, C**) Confocal microscopy image of rhodamine/phalloidin labeled F-actin (green) and DAPI-labeled cell nuclei (blue) showing aspects of the structure of the ovarioles (peritoneal sheath removed) at the time they were used for HEX 70A detection. The actin-rich polyfusomes (arrowheads in B) are seen in the center of the cystocyte rosettes in the upper region of the germarium. Ring canals derived from polyfusomes (arrows in B and C) are apparent in the lower region of the germarium shown in B and in higher magnification in C. (**D**) Confocal microscopy of an ovariole (upper portion of the germarium) stained with DAPI. (**E**) The same ovariole showing foci of HEX 70a detected with anti-HEX 70a/Cy3 (red). (**F**) The merged D and E images. The insert in F shows a “control” ovariole (upper portion of the germarium) incubated with the pre-immune serum and subsequently stained with Cy3/DAPI. Arrowheads in D-F show nuclei of germline cells. Arrows in D–F point to nuclei of follicle cell precursors. In all figures, the upper portion of the germarium is oriented upward.

Ovary sections of a pharate-adult worker show the basic structure of an ovariole stained with methylene blue and basic fuchsin (Figure 1A), the actin array visualized through rhodamine/phalloidin staining (Figures 1B, C), and foci of HEX 70a immunodetected with anti-HEX70a/Cy3 (Figures E, F). DAPI was used to highlight ovarian cell nuclei and to make ovariole visualization easier (Figures 1B, C, D, F). At this initial stage of pharate-adult development (∼1 day after pupal ecdysis), each ovariole consists of a distal terminal filament (not shown) and a proximal germarium. In the germarium the germline cells, or cystocytes, are beginning to be arranged in rosette-like structures (circle in Figure 1A). Each rosette is a cystocyte clone derived from a single cystoblast (oogonium) and will give rise to a single oocyte and the accompanying trophocytes, or nurse cells. To better visualize the structure of the ovariole at this developmental stage (early pharate-adult) we used rhodamine/phalloidin for detection of F-actin, and DAPI to stain the ovarian cell nuclei. In the upper region of the germarium (upper part of Figure 1B) we could visualize the dense actin complex typical of the polyfusomal region in the center of each cystocyte rosette (arrowheads in Figure 1B). In the lower region of the germarium (lower part of Figure 1B and Figure 1C) the polyfusomes were converted into ring canals (arrows in Figures 1B, C) that allow communication among the germline cells, i.e., among the cell destined to be the oocyte and its associated nurse cells. Ovarioles characterized by such structural arrangements, as detailed in Figures 1A–C, were prepared for HEX 70a detection with anti-HEX 70a/Cy3. Figure 1D shows the upper region of the germarium of an ovariole stained with DAPI. Figure 1E illustrates the same ovariole region where foci of HEX 70a can be seen (merged image is shown in Figure 1F). The insert in Figure 1F represents a control ovariole incubated with pre-immune serum and stained with DAPI and Cy3. Comparison among Figures 1D–F revels that HEX 70a is localized in the nuclei of the germline cells (cystocytes), in close association with chromatin (arrowheads in Figures 1D–F). Presumptive follicle cells (somatic cells) are not clearly evident at this stage, but were tentatively indicated by arrows in Figures 1D–F. Like the germline cell nuclei, the somatic cell nuclei show HEX 70a foci.

### Colocalization of EdU and HEX 70a in the ovarian cell nuclei of pharate-adult workers

**Figure 2:**
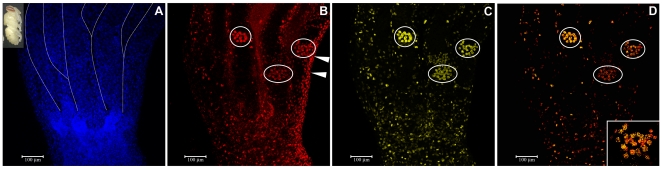
Colocalization of anti-HEX 70a/Cy3 and EdU/Alexa Fluor in the ovary of a pharate-adult worker (∼2 days after pupal ecdysis: seen at the upper left corner of the figure). (**A**) DAPI-stained ovarian cell nuclei (shown in blue). For clarity, the ovarioles are delineated by dashed lines above the basal portion of the ovary. Only the germarium region is seen in each ovariole. (**B**) HEX 70a foci detected with anti-HEX 70a/Cy3 (red). (**C**) EdU/Alexa Fluor foci in S-phase ovarian nuclei (yellow). (D) Merged B and C images. Circles in B–D emphasize groups of cystocytes (in the germarium region) showing double labeling (anti-HEX 70a/Cy3 and EdU/Alexa Fluor). The group of cystocytes encircled at the most right position in D is shown in higher magnification in the insert. Cell nuclei on the right margin of the ovary in B (arrowheads) show HEX 70a/Cy3 but not EdU/Alexa Fluor labels.

EdU is a nucleoside analog of thymidine that incorporates into DNA during the S-phase of the cell cycle, thus allowing the detection of DNA replication for cell division when coupled to a dye (Alexa Fluor 594). EdU was injected in early pharate adults (∼1 day after pupal ecdysis). The ovaries were dissected after 24 h and prepared for confocal microscopy. Figures 2A–D show confocal images of one of these ovaries. In Figure 2A the DAPI-staining highlighted the cell nuclei in the base of the ovary and in its constituent ovarioles. Only the germarium region is shown in each ovariole. Figure 2B revealed intranuclear HEX 70a/Cy3 foci spread throughout the ovary. By comparing Figures 2A and 2B we identified regions of DAPI-stained nuclei in the ovarioles (germarium) without HEX 70a/Cy3 foci. Therefore, HEX 70a is not present in every ovarian nuclei. Figure 2C revealed EdU incorporation in S-phase nuclei. In a comparative analysis, the Figures 2B, C and the merged image seen in Figure 2D revealed that the nuclei labeled with EdU/Alexa Fluor also show HEX 70a/Cy3 labels, suggesting that HEX 70a may be somehow involved in the S-phase events leading to cell proliferation in ovarioles. However, HEX 70a has a nuclear localization even in cells outside the S-phase, since the overlap between HEX 70a/Cy3 and EdU/Alexa Fluor labels is not complete: for example, the nuclei showing HEX 70a immunofluorescence at the right margin of the ovary in Figure 2B do not show EdU fluorescence (Figures 2C, D).

### Expression of HEX 70a in ovarioles of egg laying queens

**Figure 3:**
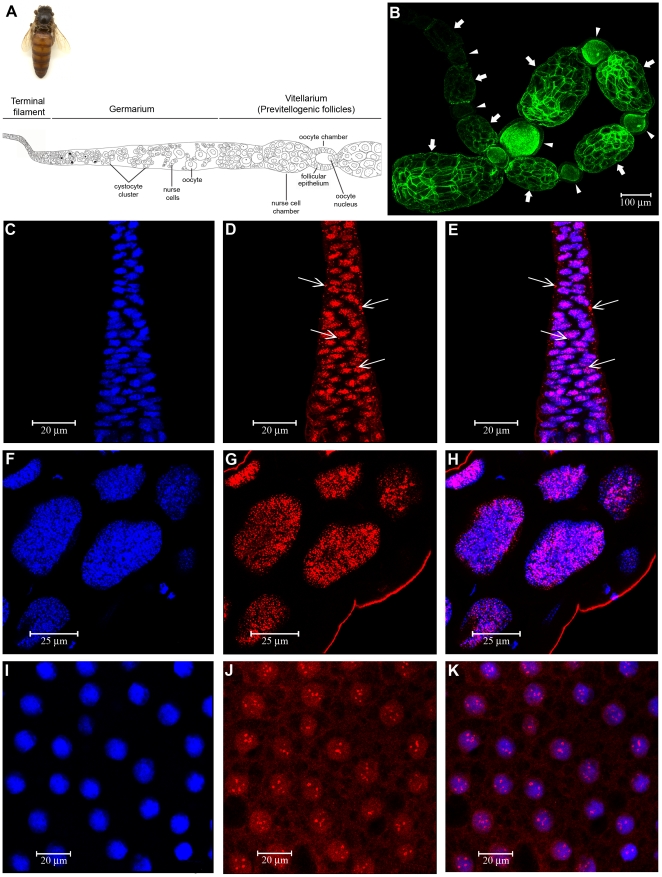
Immunolocalization of HEX 70a in the queen ovariole. (**A**) Schematic representation of an ovariole of an egg laying queen (seen at the upper left corner): only the terminal filament, the germarium and early follicles initiating previtellogenic growth in the upper region of the vitellarium are shown in A. Confocal microscopy images: (**B**) Part of an ovariole showing the middle and lower regions of the vitellarium labeled with rhodamin/phalloidin (green) to highlight F-actin. The arrows and arrowheads show developing nurse cell- and oocyte- chambers, respectively. (**C–E**) the terminal filament (the lower region is oriented downward) shows HEX 70a foci in the nuclei (D, E) and in cytoplasm (arrows in D, E). (**F–H**) Nurse cell nuclei in the nurse cell chamber (lower region of the vitellarium as indicated by arrows in B). (**I–K**) Follicle cell nuclei covering an oocyte at the lower region of the vitellarium (as indicated by arrowheads in B). (**C, F, I**) DAPI-stained cell nuclei (blue); (**D, G, J**) anti-HEX 70a/Cy3-staining for HEX 70a detection (red) and (**E, H, K**) merged images.

HEX 70a foci were also detected in ovarioles dissected from adult queens. Figure 3A shows a schematic representation of an ovariole of an egg-laying queen. The ovariole consists of a narrow distal region, the terminal filament, an intermediate region, or germarium, and a proximal region, the vitellarium. The terminal filament contains typical coin-shaped somatic cells and putative germline stem cells [42]. Cystocyte clusters are observed in the upper region of the germarium, and in the lower region there are growing oocytes associated with the polyploid nurse cells. In the upper region of the vitellarium (Figure 3A), nurse cell and oocyte chambers forming the pre-vitellogenic follicles are visible. The lower region of vitellarium is the largest region of the ovariole (shown in Figure 3B) and consists of a sequence of growing oocytes involved by a layer of follicle cells (arrowheads) interspersed with nurse cell chambers (arrows). In this region, the oocyte reaches its maximum size, the nurse cells collapse, the chorion is formed and the egg is finally released into the oviduct.

Figures 3C–E shows the lower region of the terminal filament. In this region, HEX 70a is strongly associated with cell nuclei, but foci of HEX 70a in the cytoplasm of filament cells were also noticed (Figure 3D, E, arrows). HEX 70a was also localized in the nuclei of the nurse cells (Figures 3F–H), as well as in the nuclei of the somatic follicle cells (Figures 3I–K), which cover the oocyte. In both cell types, HEX 70a has exclusively an intranuclear localization, but with a very distinct pattern of foci size and distribution. HEX 70a foci are small and scattered all over the nuclei of the polyploid nurse cells and are larger and concentrated in defined nuclear areas in the proliferating follicle cells.

### Effect of anti-HEX 70a injection on ovariole width and cuticle sclerotization

**Figure 4:**
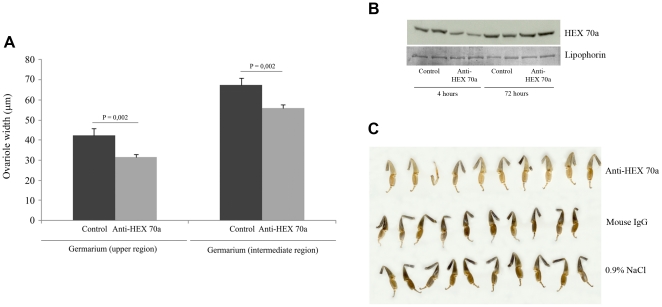
Effect of HEX 70a depletion on queen ovary growth and worker cuticle formation. (**A**) Width of the ovarioles of queens injected with anti-HEX 70a in 0.9% NaCl or saline vehicle only. Measurements were made in two regions of the germarium of 120 ovarioles, 60 of them dissected from 3 anti-HEX 70a injected queens (20 ovarioles per queen), and 60 from 3 control queens. Measurements obtained from bees injected with the antibody, or the antibody vehicle only, were compared using Two-Way ANOVA and the post-hoc Holm-Sidak multiple comparison test (Jandel SigmaStat 3.1 software, Jandel Corporation, San Rafael, CA, USA). (**B**) Western blot levels of HEX 70a in the hemolymph samples of workers at 4 and 72 h after injection with anti-HEX 70a or saline vehicle only (control). The levels of the ∼200 kDa lipophorin in the same samples were used as loading control. (**C**) Hind legs of workers injected with anti-HEX 70a in 0.9% NaCl, in comparison to workers injected with mouse IgG in 0,9% NaCl, or those of the 0.9% NaCl injected group.

To strengthen the hypothesis that HEX 70a is involved in ovariole cell proliferation we injected 24 h-queen pupae with anti-HEX 70a (diluted in 0.9% NaCl) and measured the width of the ovarioles soon after the adult ecdysis, under the expectation that the specific antibody would reduce HEX 70a activity and, thus, result in smaller ovarioles. Figure 4A shows that the antibody injection significantly hampered ovariole growth (p = 0.002) in comparison with control queens injected with the vehicle only.

In parallel, 24 h-worker pupae were also injected with anti-HEX 70a and the effect of this antibody on the hemolymph HEX 70a levels was examined. Western blots revealed a reduction of 54% (estimated by densitometric assessment in arbitrary units obtained from HEX 70a bands normalized to the ∼200 kDa lipophorin loading control) in the levels of HEX 70a 4 h after injection of the antibody, followed by recovery to normal levels within 72 h (Figure 4B). Given that HEX 70a is an arylphorin, and as such, it may represent a source of aromatic amino acids for cuticle formation, we also checked the progress of pigmentation and sclerotization in anti-HEX 70a-injected workers, comparing them to two control groups, injected with mouse IgG or only with the antibody vehicle. Anti-HEX 70a injection produced a drastic effect on cuticle formation. This effect was more evident in the cuticle of the hind legs that were not fully pigmented and sclerotized. In anti-HEX 70a-treated bees, the hind leg cuticle is clearer and softer than in the control groups (Figure 4C). Taken together, the data shown in Figure 4 are consistent with the proposed participation of HEX 70a in ovariole cell proliferation, confirmed that the antibody is effective in reducing HEX 70a levels, and furthermore, confirmed that HEX 70a is a genuine arylphorin with a role in cuticle formation (in addition to being a nuclear protein in the gonads).

### Expression of HEX 70a in the testes

**Figure 5:**
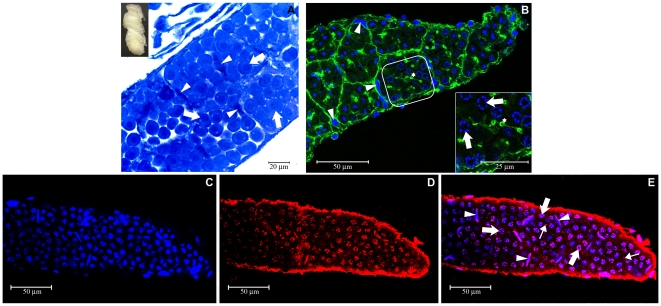
Immunolocalization of HEX 70a in the testioles of drone pupae (1 day after pupal ecdysis; developmental stage shown at the upper left corner). (**A**) Light microscopy section of a testiole stained with methylene blue/basic fuchsin showing a region containing groups of cystocytes (spermatogonia) (arrows) involved by somatic cells (somatic cell nuclei pointed by arrowheads). (**B**) Confocal microscopy image showing rhodamine/phalloidin labeled F-actin (green) and DAPI-labeled cell nuclei (blue); somatic cell nuclei are pointed by arrowheads; insert shows a magnified image of a cyst containing cystocytes (cystocyte nuclei pointed by arrows) and ring canals (asterisks). (**C–E**) Confocal microscopy images of a testiole from a drone taken at the same developmental phase, showing (**C**) DAPI-stained cell nuclei, (**D**) foci of HEX 70a detected with anti-HEX 70a/Cy3 (red), and (**E**) the merged C and D images. In Figure 1E the thick arrows show germ cell nuclei, the thin arrows show HEX 70a foci in the cytoplasm and the arrowheads show somatic cell nuclei.

HEX 70a was also detected in the germ and somatic cells of developing testes. Figure 5A shows a cross section of the upper portion of a testiole dissected from a drone pupa (1 day after pupal ecdysis). In this region we could observe cysts, i.e., groups of germ cells (cystocytes or spermatogonia: arrows in Figure 5A) housed within a somatic cell envelope (somatic cell nuclei pointed by arrowheads in Figure 5A). Confocal microscopy on rhodamine/phalloidin-labeled F-actin (green) and DAPI-labeled cell nuclei (blue) (Figure 5B) highlighted the structure of this region of the testiole. F-actin is an abundant component of the somatic cell cytoplasm, and is also present in the ring canals (asterisks in Figure 5B) that enable mutual communication for the germ cells. Comparison of Figures 5C–E revealed foci of HEX 70a mainly in the nuclei of the germ cells (thick arrows in Figure 5E) and somatic cells (arrowheads in Figure 5E), but also dispersed in the cytoplasm of the germ cells (thin arrows in Figure 5E). The small volume of cytoplasm in the somatic cells impairs the accuracy in identifying possible cytoplasmic HEX 70a foci in the confocal images.

**Figure 6:**
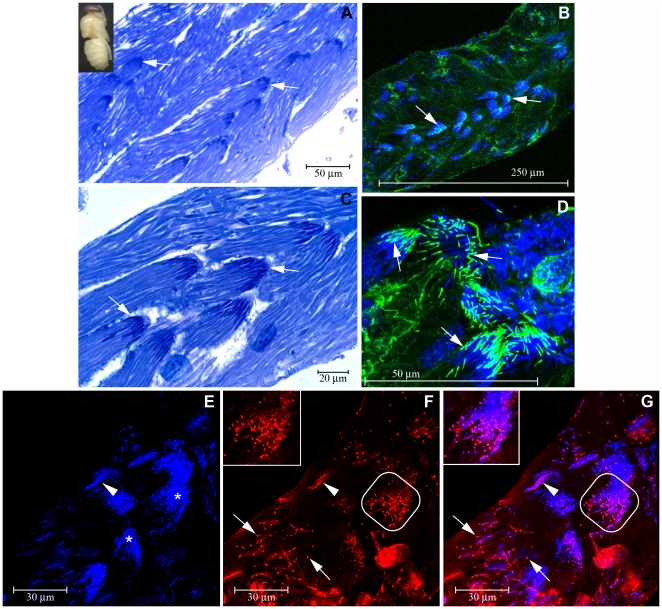
Immunolocalization of HEX 70a in the testioles of pharate-adult drones (∼6 days after pupal ecdysis; developmental stage shown at the upper left corner). (**A, C**) Light microscopy of the testiole stained with methylene blue/basic fuchsin. Syncytial cluster of spermatids are evident (arrows). (**B, D**) Confocal microscopy images showing rhodamine/phaloidin labeled F-actin (green) and DAPI-labeled cell nuclei (blue). The association of actin with spermatid heads in the syncytial clusters is evidenced in B (arrows) and in the similar and amplified D image (arrows). (**E–G**) Confocal microscopy showing (**E**) DAPI-stained nuclei in the syncytial cluster of spermatids (asterisks) and in a cyst somatic cell nuclei (arrowhead); (**F**) HEX 70a foci detected with anti-HEX 70a/Cy3 (red) at the posterior end of the spermatid nuclei in the syncytial cluster (shown in higher magnification at the upper left side): arrows point to individualized spermatozoa and the arrowhead points to a cyst somatic cell nuclei. (**G**) The merged E–F images showing the amplified syncytial cluster of spermatids (insert), individualized spermatozoa (arrows) and a cyst somatic cell nuclei (arrowhead).

Sections of the lower region of testioles dissected from drones at an intermediate phase of the pharate adult development (∼6 days after pupal ecdysis) showed syncytial clusters of elongating spermatids (Figures 6A, C, arrows). Actin cones were seen assembled around the tip of the spermatid nuclei (Figures 6B, D, arrows). Figure 6E shows DAPI-stained nuclei of spermatids in syncytial clusters (arrows) and of somatic cells (arrowheads). In Figure 6F, which is a preparation stained with anti-HEX 70a/Cy3, and in the merged image (Figure 6G) we could verify that HEX 70a was strongly localized to the posterior extremity of the spermatid nuclei (Figures 6F, G inserts), as well as in the nuclei of individualized spermatozoa (arrows in Figures 6F, G) and somatic cells (arrowheads in Figures 6F, G).

## Discussion

### HEX 70a in oogenesis and spermatogenesis of the honey bee

Herein we show that the honey bee HEX 70a is localized in the nuclei of ovarian and testis cells, thus implying in a yet undescribed role for this hexamerin. In its native structure, HEX 70a is an oligomer (data not shown). Similar to other proteins, HEX 70a may be acting in the nucleus in the monomeric form, as recently reported for royalactin, a 57 kDa monomer that functions as a caste determining factor in the honey bee. Royalactin forms the oligomere MRJP1, a member of the Major Royal Jelly Protein family, which is present not only in royal jelly secreted by the worker hypopharingeal glands, but also in hemolymph and other tissues of the honey bee [43].

HEX 70a fulfills all the criteria established for classification as a storage hexamerin. It has the three canonical hemocyanin domains (N: PF03722.5, M: PF00372.10 and C: PF03723.5 - Pfam database, [44]), which are typical of all hexamerins. It is massively synthesized by the fat body during the larval feeding stage and abundantly stored into larval hemolymph, remains in high quantity in pupal and early pharate-adult hemolymph, and subsequently becomes less abundant [23], [39]. This feature is in conformity with the role in providing amino acids for pupal and pharate adult development, just like the other hexamerins. Furthermore, it contains a high proportion (18.2%) of aromatic amino acids, which makes it a member of a subclass of hexamerins, the arylphorins. HEX 70a is likely used for adult cuticle construction. As demonstrated herein, the inactivation of HEX 70a *in vivo* by injecting anti-HEX 70a into worker pupae visibly hampered the process of adult cuticle formation. Interestingly, the experimental decrease in HEX 70a in hemolymph provoked through antibody-injection was sufficient to affect cuticle formation, despite the presence of another arylphorin, HEX 70b, in hemolymph at this stage [40]. This indicates that HEX 70a, or the amino acids derived from its hydrolysis, have essential participation in cuticle formation.

Previous experimental evidence in our laboratory had already indicated that HEX 70a is a multifunctional protein. By means of semiquantitative and quantitative RT-PCR and Western blot analysis using anti-HEX 70a, we could show that the fat body is not the only site of HEX 70a production, as the transcript and the corresponding protein subunit were also detected in developing gonads of workers, queens and drones, suggesting roles in ovary differentiation and testes maturation. HEX 70a transcripts and protein subunits were also detected in the ovaries of adult queens (but not in the worker bee hypopharyngeal glands) [23].

Following up on this question, the immunodetection of HEX 70a in the gonads now evidenced an association of this protein with nuclei of germline and somatic cells. Such localization was completely unexpected for a storage protein, implying regulatory or structural roles in the nuclei. The nuclear colocalization of HEX 70a with the S-phase marker EdU furthermore indicated that HEX 70a may play a role in DNA replication for cell proliferation or polyploidization. However, there are also ovariole cell nuclei showing HEX 70a immunofluorescence, but not EdU fluorescence (the reverse was not observed). This does not exclude a possible HEX 70a role in cell proliferation, but may indicate that HEX 70a does not have an exclusive role in the S-phase of the cell cycle, or that the stability of the protein within the nuclei is not restricted to the S-phase.

The hypothesis that HEX 70a is involved in cell proliferation received support from experiments where anti-HEX 70a antibody was injected into queen pupae, revealing negative effects on ovariole enlargement, which likely occurs via cell proliferation. Consistent with this hypothesis, HEX 70a was localized in the nuclei of the cystocytes in the ovaries of early pharate-adult workers. Cystocytes are mitotically active, as shown here by EdU labeling, and through BrdU (5-bromo-2′deoxy-uridine) labeling [42]. Each cystocyte proliferates to form a clone of about 48 or more cells [45], [46] which is arranged as a rosette and contains a germline-specific organelle, the polyfusome [47]. Actin was shown to be a prominent fusome marker in the center of the rosettes [48], [49]. Only later in development will one cystocyte in each rosette enter meiosis and begin to grow and then become morphologically distinguishable from the nurse cell-destined cystocytes. As the oocyte differentiates, the rosettes are gradually transformed into initial follicles, with the fusomes being converted into the ring canals that connect the developing oocyte with the nurse cells, and the nurse cells with each other. Each growing oocyte/nurse cell cluster becomes surrounded by somatic follicle cells and will be partitioned into an egg chamber, where oogenesis and vitellogenesis proceed, and a trophic chamber (or nurse cell chamber) [42], [46], [50]. Whilst this is the common pattern in queens, progressive oogenesis in workers it will only take place if they are released from the repressor effect of queen pheromone [51].

Different from the oocyte, which enter meiosis and remains transcriptionally silent, nurse cells undergo a series of endomitotic cycles [46], [52]. This characteristic, typical of the meroistic ovary, is an evolutionary strategy to increase the synthesis of material and organelles at a high rate during oogenesis, and export them to the growing oocyte through the ring canals [53], [54]. During oogenesis of the honey bee, the somatic follicle cells become a thick epithelium around the growing oocyte and a flattened cell layer around the joined nurse cells [55]. To account for the intense oocyte growth during oogenesis and vitellogenesis, the follicle cells that surround the oocyte must undergo several rounds of mitotic divisions. Unpublished data from our laboratory (Macedo LMF, personal communication) documented the significant increase in follicle cell number in the growing follicles of the honey bee. Consistent with a role in DNA replication, HEX 70a was localized in the polyploid nuclei of nurse cells and in the proliferating follicle cells covering the growing follicle in queen ovarioles. The pattern of HEX 70a foci in the nucleus, however, is distinct for nurse and follicle cells, perchance reflecting their respective physiological status. HEX 70a was also localized in the terminal filament cells where mitotically active BrdU labeled nuclei, probably stem germline cell nuclei, were demonstrated by Tanaka and Hartfelder (2004) [42]. Interestingly, only in this ovariole region we were able to distinguish HEX 70a foci in the cytoplasm in addition to the nuclear focal spots. We were unable to localize HEX 70a in the nuclei of meiotic oocytes.

Intranuclear foci of HEX 70a were also detected in the germ and somatic cells of the male gonad during its early and late development. Unambiguous cytoplasmic foci of HEX 70a were observed only in the earlier stages of testis development and in the terminal filament of the ovarioles. As spermatogenesis and oogenesis progresses the foci of HEX 70a become exclusively intranuclear.

In newly-ecdysed drone pupae, the clusters of dividing secondary spermatogonia, also termed cystocytes, become enveloped by actin-rich somatic cells and in the interior of these cyst capsules they develop in spermatocytes, which then initiate the meiotic division [52], [56]. Within the cyst, the germ cells remain connected by cytoplasmic bridges, the ring canals, similar to what is seen in the ovarioles. Thus, the presence of HEX 70a in the male cystocyte and somatic nuclei may also be tentatively associated to cell proliferation. However, as discussed below, HEX 70a was in addition detected in the nucleus of the non-proliferating spermatids and individualized spermatozoa.

In the honey bee drone, the entire process of spermatogenesis, from the undifferentiated male germ cell to the formation of the motile sperm occurs during postembryonic development and is essentially concluded before emergence of the drone from the brood cell. The spermatozoa then migrate from the testioles to the seminal vesicles where they are stored for a few days before copulation during the nuptial flight [45]. Spermatogenesis in the honey bee is distinguished by (1) an atypical meiosis (drones are originated from haploid eggs) during which the spermatocytes remain interconnected by cytoplasm bridges, (2) formation and subsequent elimination of supernumerary centrioles in association with the first meiotic division, and (3) unequal division of the secondary spermatocyte [57].

During spermatogenesis in *Drosophila melanogaster*, a cyst of 64 syncytial spermatids derived from a single germ cell precursor elongates as the axonemes of the sperm tails are formed. These syncytial spermatids become finally separated into individual sperm in a process named individualization, which occurs simultaneously for all 64 spermatids. Actin polymerization is important for individualization, and this process is mediated by cones of actin that assemble around each sperm nucleus. Actin cones acquire triangular shape and move away from the sperm nucleus, causing the formation of the cystic bulge, the residual body which contains cytoplasm and organelles that will be discarded. This process ultimately leads to the transition from syncytial spermatids to individualized spermatozoa [58].

During spermiogenesis of the honey bee, we could detect foci of HEX 70a at the syncytial spermatid nuclei, posteriorly to the assembled actin cones, and also in the nuclei of individualized spermatozoa. The presence of HEX 70a in the nuclei of differentiating spermatids and in the spermatozoa certainly cannot be associated to cell proliferation, but suggests a novel, distinct role for this protein also during the spermiogenesis. This is a completely novel finding concerning a hexamerin function that requires further investigation.

### Hexamerins – more than just storage proteins for metamorphosis

Almost 20 years ago it was demonstrated that fat body tissue or fat body extracts (from lepidopteran species) were efficient in stimulating *in vitro* proliferation of larval midgut stem cells of some lepidopterans [59]–[61]. Curiously, the fat body factor that induced cell proliferation was later identified as being a 77 kDa arylphorin subunit (α-arylphorin) [62]. Purified α-arylphorin stimulated midgut stem cell proliferation at a very low concentration, which excludes a simple nutritional effect. Experiments using BrdU labeling confirmed that arylphorin induces DNA synthesis. The mitogenic-stimulating activity of arylphorin was also observed *in vivo* in insects that showed increased growth rates after being fed on artificial diets containing arylphorin [62]–[64]. Therefore, experimental approaches very distinct from those utilized herein, have led to the same conclusion, i.e., that arylphorins have a role in cell proliferation.

Our results brought to light entirely unsuspected roles for a storage protein. The presence of HEX 70a in the nuclei of germline and somatic cells in ovaries and testes suggests function in regulation, structural nuclear organization and/or cell proliferation. These gonadal functions of a larval storage protein are novelties that clearly deserve further investigation.

## Materials and Methods

### Bee sampling

Africanized honey bee workers and drones in different developmental stages (pupal, pharate-adult and adult) were collected from hives maintained at the apiary of the University of São Paulo in Ribeirão Preto, Brazil. Queens were reared according to standard apicultural methods. Some queens were collected soon after pupal ecdysis and some were collected at emergence and introduced in dequeened hives to be collected later, after mating and the onset of egg laying activity. Pupae and pharate-adults were staged according to the criteria established by Michelette and Soares (1993) [65] (workers), Tozetto *et al.* (2007) [66] (drones) and Rembold *et al.* 1980 [67] (queens), which are based on the progress of eye coloration, from white to dark-brown, and absence or presence and grade of exoskeleton tanning. Before dissection, adult bees were anesthetized with gaseous nitrogen.

### Conventional light microscopy

Gonads were dissected and briefly rinsed in Ringer saline (NaCl 0.17 M, KCl 0.01 M, CaCl_2_ 0.003 M) and kept for 24 hours in cold (4°C) fixative (4% paraformaldehyde in 0.1 M phosphate buffer, pH 7.3), dehydrated in a graded ethanol series and then embedded in methacrylate resin (Historesin, Leica). Sections of 4 µm thickness were stained with methylene blue and basic fuchsin and mounted in Entellan (Merck) to be examined and photographed using an Axioskop II photomicroscope (Zeiss).

### Confocal microscopy

#### F-actin and nuclei staining

After dissection in phosphate buffered saline (PBS_1_:137 mM NaCl, 2.7 mM KCl, 10 mM Na_2_HPO_4_, 1.7 mM KH_2_PO_4_, pH 7.4), ovaries and testes were cleaned as much as possible of trachea and immersed in a honey bee-specific tissue culture medium [68] for separating individual ovarioles and testioles and removal of the peritoneal sheath. Ovarioles and testioles were then fixed for 30 min in 240 µL PBS_1_, 200 µL 37% formaldehyde (Merck), 8 µL Triton X-100 (Sigma) and 2 µL rhodamine-phalloidin 1∶100 v/v (Invitrogen). After being washed twice in 0.2% Triton X-100 in PBS_1_ (0.2% TPBS) and 0.1% rhodamine -phalloidin for 20 min each, a third 20 min-wash was done in 0.2% TPBS without rhodamine -phalloidin. Ovaries and testioles were then incubated for 5 min in DAPI (4′,6-diamidino-2-phenylindole) 1∶8000 v/v (Sigma) in 0.2% TPBS and then rinsed five times in 0.2% TPBS. Slides were mounted in glycerol 80% (Merck) and examined under a Leica TCS-SP5 confocal microscope (Leica Microsystems).

#### HEX 70a immunolocalization

A custom-made polyclonal anti-HEX 70a specific antibody (Affinity BioReagents, Golden, CO, USA) was produced from the sequence SYKMHQKPYNKD of the HEX 70a subunit predicted from the fully sequenced cDNA [23]. This antibody was used in whole mount preparations of ovarioles and testioles from bees in different developmental stages. The pre-immune serum was used as negative control.

Ovarioles and testioles were fixed for 20 min in 4% paraformaldehyde in PBS_1_, permeabilized with 0.1% Triton X-100 in PBS_1_ (0.1% TPBS) for 15 min (five washes), blocked with 1% BSA for 30 min and incubated in 5% normal goat serum for 30 min. Ovarioles and testioles were incubated with anti-HEX 70a at a concentration of 1∶50 in 0.1% TPBS, 1% BSA and 5% normal goat serum for 16 h at 4°C. This was followed by five washes of 20 min in 0.1% TPBS, blocking with 1% BSA for 20 min (two washes) and incubation in 5% normal goat serum for 30 min. A Cy3-conjugated goat anti-rabbit antibody (Sigma, 1∶200 dilution) was added to the preparations, which were incubated for 2 h at room temperature. Ovaries and testioles were then incubated in DAPI (4′,6-diamidino-2-phenylindole) 1∶8000 v/v (Sigma) in 0.1% TPBS for 5 min and then rinsed five times in 0.1% TPBS. Slides were mounted in glycerol 80% (Merck) and examined under a Leica TCS-SP5 confocal microscope (Leica Microsystems).

#### EdU and HEX 70a colocalization

Newly ecdysed worker pupae (Pw phase) collected from hives and kept in an incubator at 34°C and 80% relative humidity for 24 h were injected with 1 µl of a 40 µM 5-ethynyl-2′deoxyuridine (EdU, Click-iT™ EdU Imaging Kits – Invitrogen) solution in Ringer saline. The injection was administered into the abdominal hemocoel. After 24 h the injected bees were dissected for extraction of the ovaries. The ovaries were fixed in 3.7% formaldehyde in PBS_1_ for 30 min and subsequently transferred to the Click-iT™ EdU Imaging Kits reaction mixture (43 µl 10X reaction buffer; 38 µl distilled water; 20 µl copper sulphate; 1.2 µl Alexa Fluor 594; 50 µl reaction buffer additive) where they remained for 30 min. The permeabilization and HEX 70a localization were performed as described above.

### Effect of anti-HEX 70a on hemolymph levels of HEX 70a and on cuticle sclerotization

#### Treatment of workers and queens with anti-HEX 70a

Newly ecdysed queen and worker pupae (Pw phase) were collected from hives and maintained in an incubator at 34°C and 80% relative humidity for 24 h before receiving an injection of 1 µl (1 µg) of the anti-HEX 70a antibody, diluted in 0.9% NaCl, into the abdominal hemocoel. Controls received 1 µl of 0,9% NaCl or 1 µl (1 µg) of mouse IgG (ECL™ Western Blotting Analysis System, Amersham Biosciences) in 0,9% NaCl. The injected queens and workers, and their respective control groups, were maintained in the incubator until the adult ecdysis. Since HEX 70a is an arylphorin, and as such it may be implicated in cuticle formation, the progress of pigmentation and sclerotization was followed daily until adult ecdysis. Following adult ecdysis of the control worker bees, the hemolymph was collected from both worker groups (control and experimental) for Western blot analysis to attest the levels of free HEX 70a. Queens had their ovarioles dissected soon after adult ecdysis and stained with DAPI for measurement of width.

#### Western blot

The hemolymph samples from the newly ecdysed adult workers injected with anti-HEX 70a or with saline vehicle only were centrifuged at 2000×*g* for 1 min at 4°C. Total protein was quantified [69] in the supernatants and samples containing 5 µg of total protein were used for electrophoresis in denaturing conditions [70] carried out at 15 mA and 4°C using 7.5% polyacrylamide gels (100×120×0.9 mm). Following electrophoresis, the proteins were transferred to nitrocellulose membranes (ImmunBlot™ PVDF Membrane). The membranes were stained with Coomassie Brillant Blue (CBB) to check migration of hemolymph proteins and molecular mass markers (205, 116, 97.4, 66, 45 and 29 kDa, Sigma). Non-specific biding sites were blocked by incubating the membranes for 16 h with 10% non-fat dried milk in PBS_2_ (50 mM Tris, 80 mM NaCl, 2 mM CaCl_2,_ pH 8.5). HEX 70a subunits were detected by incubating the membranes for 1 h, at room temperature, with anti-HEX 70a antibody diluted 1∶5,000 in 10% non-fat dried milk in PBS_2_. The membranes were washed thoroughly in 0.05% Tween 20 in PBS_1_ (0.05% TwPBS) and subsequently incubated for 1 h in a horseradish peroxidase labeled anti-rabbit IgG secondary antibody (Amersham Biosciences), diluted 1∶12,000 in 0.05% TwPBS. After washing in 0.05% TwPBS, the detection was carried out by using the ECL System (ECL™ Western Blotting Analysis System, Amersham Biosciences). The constitutively expressed ∼200 kDa hemolymph lipophorin identified in the CBB-stained nitrocellulose membranes was used as a loading control.

#### Measurements of ovary width

Ovaries from HEX 70a antibody-injected queens and from 0.9% NaCl-injected controls were fixed in 3.7% formaldehyde in PBS_1_ during 30 min and incubated in DAPI (1∶400 dilution) in 0.1% TPBS for 5 min. After rinsing five times in 0.1% TPBS the ovarioles were mounted in 80% glycerol for analysis in a Leica TCS-SP5 confocal microscope system. Ovariole width was measured by using the software LAS AF Lite 2.4.1 (Leica Microsystems).
